# Direction and Progression of Thermal Acclimation Effects on Ciliated Protist Populations Both Depend on Direction of Thermal Change

**DOI:** 10.1002/ece3.73633

**Published:** 2026-05-05

**Authors:** Julia Bebout, Jeremy W. Fox

**Affiliations:** ^1^ School of Biological Sciences University of California San Diego La Jolla California USA; ^2^ Department of Biological Sciences University of Calgary Calgary Alberta Canada

**Keywords:** phenotypic plasticity, population dynamics, protist microcosm, thermal acclimation

## Abstract

Organisms acclimate to environmental temperatures to maintain physiological homeostasis. Acclimation can alter demographic rates, thereby affecting population dynamics. Previous research has demonstrated that acclimation can have positive or negative effects on population growth rates in variable thermal environments, depending on the amount of time acclimation takes. A clear picture of the timescale of acclimation may help identify the consequences of various frequencies of thermal fluctuations for population dynamics. However, the progression of population‐level effects of acclimation over time has not been explored. We used experimental microcosms to test the effects of acclimation on population dynamics of the ciliated protist *Colpidium striatum* in various thermal regimes. We also observed the progression of these effects over the course of acclimation. Prior acclimation to cooler conditions increased intrinsic growth rates in warm trial conditions relative to the growth rates of warm‐acclimated populations. In contrast, prior acclimation to warm conditions decreased intrinsic growth rates in cool trial conditions relative to the growth rates of cold‐acclimated populations. These results are consistent with an overcompensatory acclimation response and either the “colder is better” or “optimal acclimation temperature” hypothesis, though we cannot distinguish between these two possibilities without an intermediate acclimation temperature treatment. The observed patterns may be due to resource uptake dynamics and/or increased stress at high temperatures with increased exposure duration. The progression of these effects over the course of acclimation also differed in trajectory, and perhaps duration, for populations acclimating to warmer versus cooler conditions. Differences in the effects and progress of thermal acclimation depending on the direction of thermal change suggest that different physiological mechanisms may be driving acclimation to warmer versus cooler conditions.

## Graphical abstract

Previous research has demonstrated that acclimation can have positive or negative effects on population growth rates in variable thermal environments, depending on the amount of time acclimation takes. A clear picture of the timescale of acclimation may help identify the consequences of various frequencies of thermal fluctuations for population dynamics. We found that the progression of acclimation effects over time differed in trajectory and timing for populations acclimating to warmer versus cooler conditions.

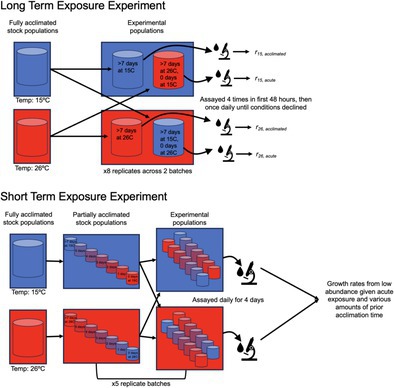

## 1 Introduction

When organisms experience a change in temperature, thermal acclimation is one way to adjust to the new conditions, maintaining physiological homeostasis and fitness. Thermal acclimation is a type of physiological, reversible phenotypic plasticity, the ability of an organism to express variation in its traits within its lifetime in response to environmental change. Acclimation occurs when organisms sense a temperature change and signal cells to generate a molecular response (Angilletta 2009). As a result, organisms accustomed to a given temperature experience altered demographic rates when placed in new conditions (Layden et al. 2022; Luhring and Delong 2017). Eventually, once the acclimatory response to temperature change has been fully executed, the organisms are accustomed to the new conditions and their demographic rates may match those of organisms that have previously acclimated to the new conditions.

These transient changes to demographic rates can affect population dynamics, particularly when temperature fluctuates over time. Acclimation is not instantaneous and can occur slower than temperature changes, giving the organism transient traits that depend on both their past and present environments (Kremer et al. 2018; Dupont et al. 2024; Burton et al. 2022). When acclimation occurs slower than temperature changes, population growth rates are averaged over the temperature fluctuations, and population density does not fluctuate along with temperature. Thus, whether a population averages over or tracks environmental fluctuations depends on the relative timescales of environmental fluctuations and the population's response to them (Bernhardt et al. 2020; Jiang and Morin 2007; May 1976; Petchey 2000). Accordingly, the timing and trajectory of acclimation effects shape population dynamics in variable thermal environments. However, the range of potential effects of acclimation on population dynamics remains largely unclear for many organisms, including ciliated protists. This uncertainty, in turn, limits our understanding of dynamics at higher levels of organization (Ahme et al. 2024).

Acclimation may also have overall positive or negative effects on intrinsic growth rates, depending on the energetic cost of physiological changes. If an organism acclimates to new conditions and those conditions change again rapidly, the organism has paid an energetic price that did not yield a benefit and could leave it maladapted (Angilletta 2009). Energetic costs can arise from both sensory physiology and the process of generating new structures (DeWitt et al. 1998; Angilletta 2009). These interacting factors can give rise to a variety of acclimation responses (Havird et al. 2020). At one extreme, acclimation can overcompensate for the acute thermal response, causing a response opposite to the sign of the acute thermal response. At the other extreme, acclimation can inverse‐compensate, or exaggerate, the acute thermal response. For example, in some systems, overcompensation is observed when organisms with prior cold acclimation outperform organisms without prior cold acclimation in warm conditions (Leroi et al. 1994; Fey et al. 2021; Layden et al. 2022; Calbet and Saiz 2022; Stuczyńska et al. 2022; Huey et al. 1999). Temperature dependent resource storage has been suggested as one possible mechanism to explain the greater fitness of some acutely exposed unicellular algae: slower cell division in cool conditions may allow resources to accumulate within the cell, which are then rapidly utilized for reproduction when a cell is introduced to warmer conditions (Anderson et al. 2024; Kremer et al. 2018).

Ciliated protists inhabit shallow freshwater bodies where thermal changes may stimulate thermal acclimation. Ciliates have been observed to acclimate in laboratory experiments (Forster et al. 2013; Calbet and Saiz 2022; Stuczyńska et al. 2022). Ciliates mainly reproduce asexually, via cell division. The time between cell divisions ranges widely, from 2.4 to almost 100 h, depending on environmental conditions including temperature (Fenchel 1968). Because daughter cells inherit the physiological state of the parent cell when cells divide asexually, thermal acclimation may extend over multiple generations. Thermal acclimation of ciliate cell size can be seen mostly within 3–5 days (Forster et al. 2013), while other cell traits, like velocity and shape, seem to acclimate within several hours of a larger increase in temperature (Dupont et al. 2025). It is likely that acclimation may proceed by additional physiological changes as well, for example, expression of proteins with more optimal thermal performance. One species of ciliate, *Tetrahymena thermophila*, expresses heat shock proteins in very warm conditions which aid in protein folding and prevent denaturation due to thermal stress (Fukuda et al. 2015). Responses to temperature changes can, however, vary greatly even among different genotypes of the same protist species (Walton et al. 1995; Dupont et al. 2025).

Here, we quantified the intrinsic growth rates of protist populations with various durations of prior acclimation to the experimental conditions to investigate how intrinsic growth rates change over time as acclimation takes place. While the physiological effects of various durations of acclimation to warm or cool conditions on individuals have been investigated for a variety of species (e.g., Bay and Palumbi 2015; Enriquez and Colinet 2019; Mohapatra et al. 1987; Pero et al. 2022; Stewart et al. 2023; Weaving et al. 2022; Dupont et al. 2025), the effects on population dynamics are relatively unexplored. Using heterotrophic protist microcosms, we tested (1) the effects of thermal acclimation on population dynamics, and (2) the progression of these effects. Protist microcosms, simple artificial ecosystems with bacteria and a focal ciliated protist species, are an ideal model system for studying the effects of acclimation on population dynamics for many reasons (Altermatt et al. 2015). Protists have short generation times, making it possible to conduct replicated long‐term experiments in just a few weeks. Additionally, the small scale of these ecosystems makes them practical for studying fluctuating thermal environments using incubators, unlike larger, field‐based systems. Finally, differences observed between acclimated and acutely exposed treatments can be attributed to acclimation due to the presumably minimal genetic diversity of populations mainly reproducing asexually (Luhring and Delong 2017).

We hypothesized that:

1. Acclimation to any new temperature will initially be detrimental to intrinsic growth rates due to energetic costs of acclimation which result in slower growth and reproduction.
2. The effects of acclimation on intrinsic growth rates in new thermal environments will decline gradually over time as acclimation progresses for 3–5 days (under three generations).

## 2 Materials and Methods

### 2.1 Study Organism

We used *Colpidium striatum* as the focal ciliate species. It has a thermal optimum of ~20°C and exhibits positive intrinsic growth rates in the thermal range of 10°C–28°C (Wieczynski et al. 2021). This aerobic ciliate is found at the bottom of ponds and lakes (Sanders 2009). Prior to experiments, populations of *Colpidium* with bacteria were obtained from Carolina Biological Supply and maintained for approximately 2 months in 250 mL stock cultures at ~20°C, remade monthly, starting from low initial protist abundance.

### 2.2 Experimental Microcosm Materials and Methods

Microcosms were constructed following established methods with small modifications (Laan and Fox 2020). Culture vessels were 100 mL glass bottles, loosely screw‐capped to allow gas exchange while reducing contamination. Each microcosm had one sterile red wheat seed in 20 mL of culture medium inoculated with bacterial food sources for the focal ciliates. We prepared culture medium with 0.7 g of finely ground, dry alfalfa‐based Protozoan Pellets from Carolina Biological Supply Company and 1 L of water from a spring‐fed stream in Big Hill Springs Provincial Park, Alberta. After autoclave sterilization and inoculation, we left culture medium at room temperature for 24 h prior to construction of microcosms, allowing the bacteria to grow to high density. The bacterial strains used were isolated from protist stock cultures and maintained on agar plates at room temperature. To reduce effects of carbon limitation or metabolic waste products, every 7 days, we removed 10% of the microcosm volume and replaced it with sterile medium, replacing withdrawn volume from sampling to keep the microcosms at 20 mL.

Two experimental temperatures, 15°C and 26°C, were selected based on the temperature change for which acclimation was stimulated in phytoplankton (Kremer et al. 2018). These temperatures are above and below the thermal optimum of *Colpidium* (~20°C; Wieczynski et al. 2021) to stimulate acclimation to both conditions, rather than subjecting populations to one optimal temperature and one suboptimal temperature. Yet, due to the asymmetrical shape of thermal performance curves (Arnoldi et al. 2025), 26°C may be slightly more stressful than 15°C for *Colpidium* (Wieczynski et al. 2021), a possibility we consider in detail in the Discussion. Microcosms were grown in dark, well‐ventilated incubators and never removed for more than 10 min.

### 2.3 Long‐Term Exposure Experiment: Effects of Acclimation on Intrinsic Growth Rates

Protist microcosms were established either with prior acclimation of stock populations to the experimental temperature (15°C or 26°C) for at least 1 week or without prior acclimation (acute exposure). Acute exposure microcosms were established using stock populations acclimated to the alternate temperature; for example, microcosms at 15°C were initiated from populations previously acclimated to 26°C. Microcosms began with *Colpidium* at a known, low initial density to obtain intrinsic growth rates in each thermal acclimation treatment. All treatments were replicated eight times between two blocks, combined for analysis.

After swirling to homogenize microcosm contents, samples of 0.3 mL were removed four times over the first 48 h, then once daily until culture conditions declined (4 weeks, ~18–41 generations depending on treatment), generally after the populations reached carrying capacity. For each sample, all individuals were counted under a microscope and diluted if necessary. For the first 48 h, microcosms with zero protists in 0.3 mL were sampled at increasingly larger volumes—1, 5, and 10 mL—until protists were observed to estimate very low abundances. These larger volume samples were examined on sterile petri plates, then returned to the microcosms. All 0.3 mL samples were permanently removed from the microcosms.

To infer intrinsic growth rates for each thermal acclimation treatment, we parameterized logistic growth models using the species abundances over time. Experimental bacterivorous protist microcosm population dynamics in constant conditions can be well‐approximated by the logistic equation (e.g., Gause 1934; Vandermeer 1969; Fox 2007). We estimated intrinsic growth rate (*r*) and carrying capacity (*K*) by fitting the logistic equation to all data from a given treatment using trajectory matching, an effective approach given the low environmental stochasticity of this model system (Harrison 1995; Rosenbaum and Fronhofer 2022). To use this approach, we assumed that, due to the tightly controlled experimental conditions, all variation among replicate population abundances was sampling error, not true variation among microcosms or batches of culture medium. The culture medium, temperatures, and stock populations were all held as constant as possible within and between batches of experiments.

We conducted model fitting using the FME package in R (R v4.3.2, FME v1.3.6.3; R Core Team 2025; Soetaert and Petzoldt 2021), which iteratively tests many possible sets of parameter values and each time calculates a measure of model misfit. The optimal set of parameter values is identified as the set which reduces this model misfit as much as possible. We used a mean‐weighted model cost to prioritize fitting low abundance data, due to the Poisson distribution of count data. We compared estimates of intrinsic growth rates for acclimated and acutely exposed populations at each temperature (*r*_15,acclimated_, *r*_26,acclimated_, *r*_15,acute_, *r*_26,acute_) to observe the effects of acclimation using two‐tailed *t*‐tests.

The estimated intrinsic growth rates are not likely to be constant over the course of the experiment for acutely exposed populations (*r*_15,acute_, *r*_26,acute_), like any experiment which quantifies growth rates for such populations (e.g., Kremer et al. 2018; Fey et al. 2021). However, assuming constant parameters allowed us to use simple quantitative models rather than making complex, unfounded assumptions about the process of acclimation. Additionally, the assumption of constant parameters is acceptable for noting differences in intrinsic growth rate for two reasons. First, we expected that the process of ciliate acclimation would take several days (Forster et al. 2013), and second, the earliest experimental data is used to estimate intrinsic growth rate. Accordingly, the estimated intrinsic growth rates are of interest while estimated carrying capacities reached after several days for acutely exposed populations likely are not informative, because the estimated carrying capacity may reflect densities achieved after complete acclimation. Estimates of intrinsic growth rates should not be taken for absolute values, but rather evidence of effects from the various thermal acclimation treatments.

### 2.4 Short‐Term Exposure Experiment: Trajectory and Duration of Acclimation Effects

**FIGURE 1:**
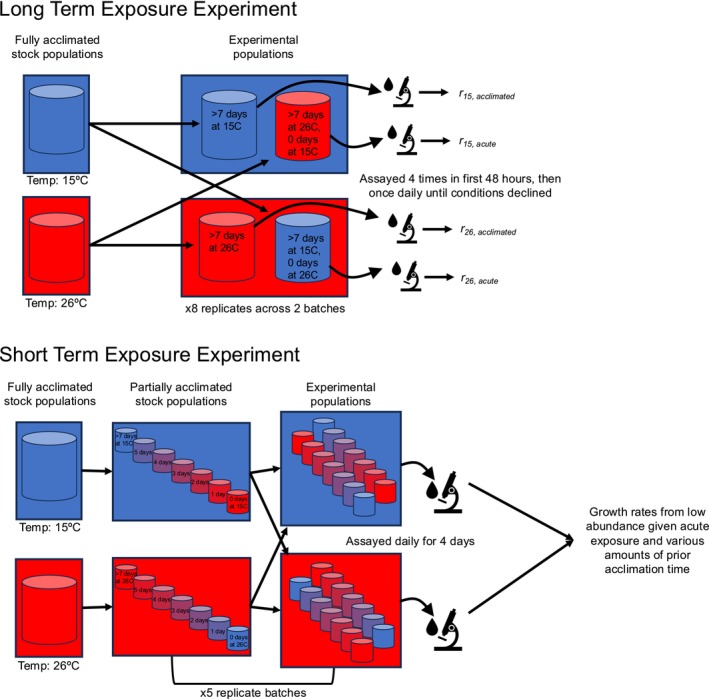
Conceptual diagram of long and short‐term exposure experiments' methods.

New protist microcosms were established as in the long‐term exposure experiment, but with either acute exposure or various lengths of time for stock populations to acclimate to the experimental temperature (15°C or 26°C) prior to microcosm establishment (Figure 1). First, seven stock populations in 15°C and 26°C were established with *Colpidium* at low initial abundance at least 10 days prior to experiments to allow the populations to reach high abundance and fully acclimate. Then, at the appropriate times, 1 (less than one generation time) to 5 days (less than about 3 generations) prior to establishing microcosms, five stock populations were moved from their acclimation temperature to the alternate temperature. The two remaining stock populations which spent more than 10 days acclimating to either 15°C or 26°C were used to test fully acclimated thermal exposure and acute thermal exposure (zero days of acclimation, more than 10 days of acclimation to the alternate experimental temperature). Using these seven stock populations, we established microcosms with *Colpidium* at a known, low initial abundance, representing all acclimation durations (0, 1, 2, 3, 4, 5, and > 10 days, 0 to > 3 generations) and experimental temperatures (15°C and 26°C). The microcosms were sampled daily for 4 days using the same procedure as the long‐term exposure experiment. These treatments were replicated five times, blocked by time, which we combined for analysis. We dropped one replicate block from analysis due to failure of the populations to grow for unknown reasons.

To estimate constant intrinsic growth rates for each acclimation time treatment, protist densities were log transformed and linearly regressed on elapsed experimental time. We used only the protist densities observed in the first 3 days of the intrinsic growth rate assay to remove any data which might reflect non‐exponential growth as the most rapidly growing populations approached carrying capacity. By observing the log transformed protist densities over time and residuals vs. fitted values, we confirmed that there was no evident density dependence in per capita intrinsic growth rate over the first 3 days of the experiment. These intrinsic growth rates were then compared across the various acclimation time treatments using linear regression (for 0–5 days of acclimation), and ANOVA with post hoc Tukey's test (for 0 to more than 10 days of acclimation). We also used the two‐lines test (Simonsohn 2018) to infer whether intrinsic growth rate had a humped or U‐shaped relationship to acclimation time. The two‐lines test identifies whether two regression lines of opposite sign may be drawn which are both independently statistically significant (Simonsohn 2018). We performed the two‐lines test on data for populations with only 0–5 days of acclimation because the populations with more than 10 days of acclimation represented various amounts of time for prior acclimation greater than 10 days.

As in the previous experiment, these estimates of intrinsic growth rates from acutely exposed populations were calculated using early experimental data before acclimation is expected to be complete. If acutely exposed populations acclimated to the experimental temperature during the 5 days of the experiment used to assay intrinsic growth rates, growth rates of acutely exposed populations and acclimated populations in the same experimental temperature would converge, making our approach conservative.

## 3 Results

### 3.1 Effects of Acclimation on *Colpidium* Growth Rates

**FIGURE 2:**
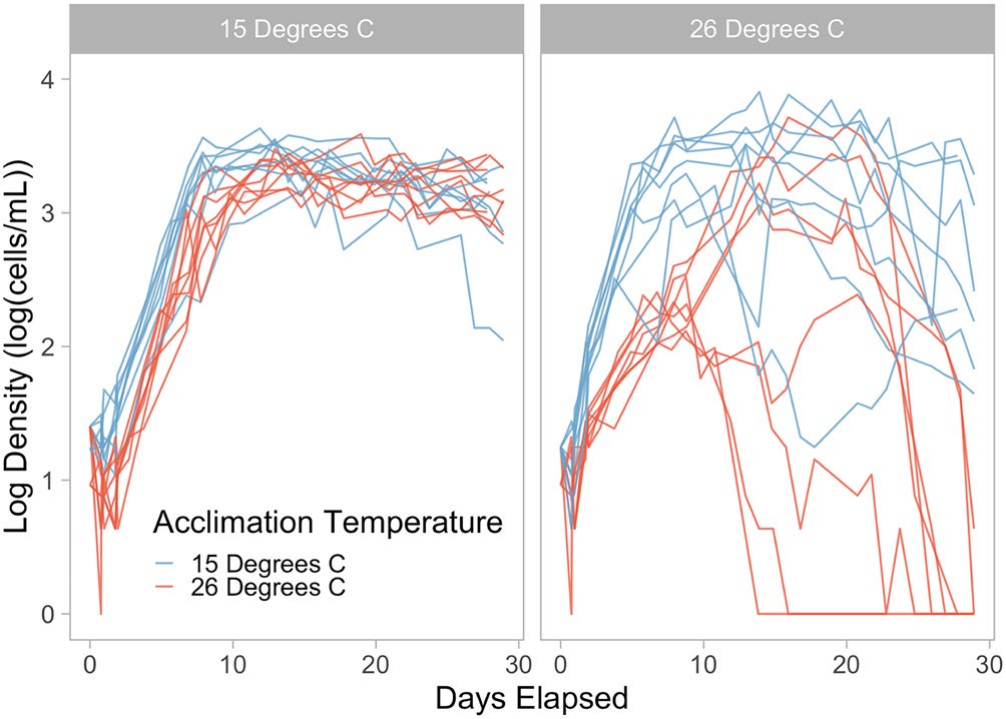
From the long‐term exposure experiment, population dynamics for *Colpidium striatum* at constant experimental temperatures of 15 (left) and 26°C (right), colored by prior > 7‐day acclimation temperature. On the y axis, densities are log base 10 transformed densities, plus one for ease of transformation.

**FIGURE 3:**
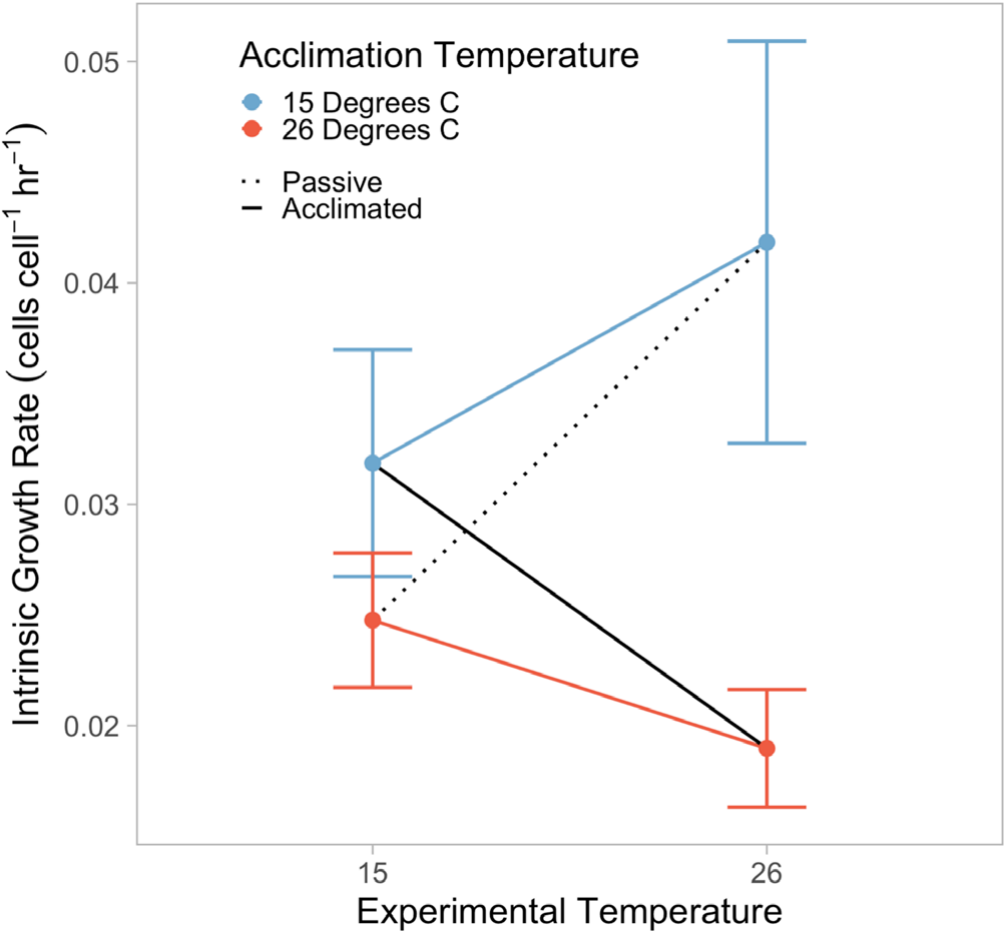
Intrinsic growth rates (*r*) estimated via the long‐term exposure experiment based on logistic model fitting of population dynamics for *Colpidium striatum* at either 15 or 26°C with or without prior acclimation, colored by acclimation temperature. Populations without prior acclimation were acclimated to the alternate temperature, that is, a population acutely exposed to 26°C was acclimated to 15°C. Error bars represent standard error for each estimate. Estimates based on *n*_15,acclimated_ = 150, *n*_26,acclimated_ = 94, *n*_15,acute_ = 140, and *n*_26,acute_ = 153. Acclimated and passive thermal response lines following Havird et al. (2020).

In the long‐term exposure experiment, *Colpidium* populations grown at 15°C with prior acclimation to 15°C grew more rapidly than populations at 26°C with prior acclimation to 26°C on average (*r*_15,acclimated_ = 0.032 ± 0.005 cells cell^−1^ h^−1^ (mean ± SE), *r*_26,acclimated_ = 0.019 ± 0.003 cells cell^−1^ h^−1^, *t =* 2.23, df = 748, *p* = 0.026; Figures 2 & 3). Cold acclimated populations grown at 26°C had significantly greater estimated intrinsic growth rates than warm acclimated populations grown at 26°C (*r*_26,acute_ = 0.042 ± 0.009, *t* = −2.416, df = 748, *p* = 0.016; Figure 3), but at 15°C, estimated intrinsic growth rates were not significantly different between populations with prior warm and cold acclimation (*r*_15,acute_ = 0.025 ± 0.003, *t* = 1.191, df = 748, *p* = 0.234; Figure 3). Some cold acclimated populations grown at 26°C appeared to exhibit two phases of rapid growth, around 0–2 days and around 8–10 days, suggesting that acclimation may have taken up to this length of time (Figure 2). Extinctions were more common at 26°C (7 of 16 microcosms) than 15°C (0 of 16 microcosms, Figure 2). Carrying capacity was significantly higher at 15°C than 26°C for acclimated *Colpidium* (*K*_15,acclimated_ = 1994 ± 158 cells mL^−1^, *K*_26,acclimated_ = 645 ± 51 cells mL^−1^, *t* = −8.137, df = 748, *p* < 0.001; Figure 2).

### 3.2 Timing and Trajectory of *Colpidium* Growth Rates During Acclimation

**FIGURE 4:**
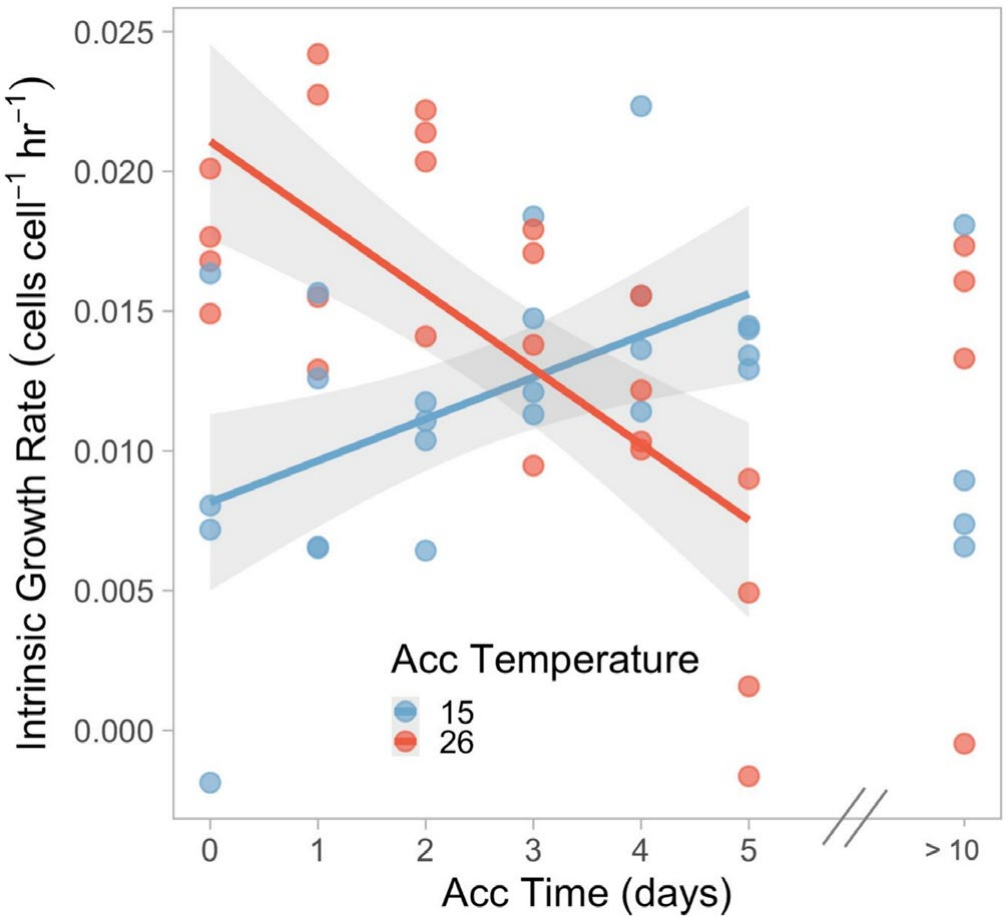
From the short‐term exposure experiment, intrinsic growth rates (*r*) for *Colpidium striatum* at experimental temperatures of 15°C and 26°C after zero to more than 10 days of acclimation prior to assay, colored by acclimation temperature. We conducted linear regression only for populations with 0 (acute exposure after prior acclimation to the other experimental temperature) to 5 days of acclimation prior to assay. Gray areas represent 95% confidence intervals.

The short‐term exposure experiment revealed that intrinsic growth rates at 15°C and 26°C follow different trajectories as populations progress from acutely exposed to fully acclimated (Figure 4). In a general linear model in which intrinsic growth rate depended on acclimation time, the experimental temperature, and the interaction of these variables, the interaction term was highly significant (*t* = −4.035, df = 52, *p* < 0.001). At 15°C, as the number of days of prior acclimation to 15°C increased, intrinsic growth rate increased linearly by approximately 0.0015 ± 0.0005 cells cell^−1^ h^−1^ day^−1^ (*t* = 2.978, df = 22, *p* = 0.007) to the acclimated growth rate over about 3–5 days (Figure 4). At 26°C, as the number of days of prior acclimation to 26°C increased, intrinsic growth rates decreased; in the first 5 days, intrinsic growth rate declined by approximately 0.0027 ± 0.0006 cells cell^−1^ h^−1^ day^−1^ (*t* = −4.88, df = 22, *p* < 0.001; Figure 4). Intrinsic growth rate after 5 days of acclimation to the experimental temperature was not significantly different from intrinsic growth rate after more than 10 days of acclimation at 26°C (*t* = −2.41, df = 21, *p* = 0.241) or 15°C (*t* = 1.11, df = 21, *p* = 0.918). Neither acclimation process for the first 5 days of acclimation was significantly U‐shaped, demonstrated by the two‐lines test.

## 4 Discussion

### 4.1 Trajectory and Duration of Acclimation Effects

Experiments revealed effects of acclimation on the population dynamics of *Colpidium striatum*. Contrary to our first hypothesis, these effects of acclimation differed depending on the direction of thermal change. These results are consistent with an overcompensation acclimation type (Havird et al. 2020) and either the “colder is better” or “optimal acclimation temperature” hypothesis, though we cannot distinguish between these two possibilities without an intermediate acclimation temperature treatment (Huey et al. 1999). Additionally, effects of acclimation on intrinsic growth rate over time depended on the direction of thermal change. Aligning with our second hypothesis, acclimation to cool conditions proceeded along a trajectory which was seemingly linear, at least when observed at daily intervals (Dupont et al. 2025), with acute intrinsic growth rate increasing to the acclimated intrinsic growth rate over about 5 days or fewer. However, the trajectory of acclimation to warm conditions was less clear. Two possibilities are consistent with our data. First, acclimation to warm conditions decreased intrinsic growth rate over time, eventually stabilizing at a low level. Second, acclimation to warm conditions requires longer than the 5 days we observed. In this case, intrinsic growth rate would initially decline but eventually recover, following a nonlinear trajectory. Because we did not expect acclimation to take over 5 days, our experiment cannot distinguish between these possibilities. However, a subtle, second phase of rapid growth around 8–10 days in some *Colpidium* populations exposed to 26°C after prior acclimation to 15°C (Figure 2) could suggest that acclimation to 26°C takes about 8 days, though this is far from conclusive.

The time periods observed here for acclimation to warmer and cooler conditions may add context to the results of a prior study which reported that dinoflagellates consistently acclimate within three generations. Franzè and Menden‐Deuer (2020) subjected dinoflagellates to gradual temperature changes and allowed them to acclimate for three generations. They found that the growth rates of these acclimated populations were the same as populations which were continuously incubated at the same temperature for 10–30 days, suggesting that complete acclimation consistently occurred in under three generations. Here, we observe acclimation time as the number of days of acclimation to a novel temperature required for a population to grow at the same rate as a population continuously reared in that temperature. Franzè and Menden‐Deuer (2020) measured acclimation time in generations, which is related to thermal performance and cannot be easily compared to our results. However, in the short‐term exposure experiment, the range of acclimation times used (0–5 days) resulted in populations which, based on observed growth rates, ranged from zero generations of acclimation to a maximum of around three generations of acclimation. Our results illustrate the complex effects of acclimation that occur within three generations of experiencing a novel temperature, and also suggest that complete acclimation to suddenly warmer conditions could possibly require more than three generations.

Our finding that acclimation can be beneficial or detrimental depending on the direction of thermal change aligns with those of many studies of diverse organisms. For algae, populations grow the most rapidly, or produce the most toxin, immediately after being switched to warm conditions, having first acclimated to cool conditions (Kremer et al. 2018; Layden et al. 2022). In bacteria, rotifers, other ciliate protist species, and insects, acclimation has similar effects, with cool‐temperature rearing benefiting various fitness metrics (Leroi et al. 1994; Luhring and Delong 2017; Zamorano et al. 2017; Klepsatel et al. 2020; Calbet and Saiz 2022; Stuczyńska et al. 2022).

### 4.2 Possible Mechanisms of Observed Acclimation Effects

Based on our findings of acclimation trajectories and times which depended on the direction of thermal change in a heterotrophic protist, we suggest that different mechanisms may be operating for acclimation to warmer versus cooler conditions in our system. Several processes might explain the linear (at least at the observed level of granularity) under 5‐day acclimation process of populations moved from warm to cool conditions. Cells may be generating new proteins with lower thermal optima, which could manifest as gradually increasing intrinsic growth rate to converge upon the acclimated state, as observed for several ciliate traits after thermal change by Dupont et al. (2025).

A possible mechanism that may explain the initially positive effect of cold acclimation in warmer conditions is resource uptake and storage. Studies of other organisms, including insects, have found that acclimation to cool temperatures was beneficial due to resource storage (Klepsatel et al. 2020; Zamorano et al. 2017). This hypothesis has also been explored empirically using algae, which contain less nitrogen and phosphorus at warmer temperatures (Layden et al. 2022; Yvon‐Durocher et al. 2017; Woods et al., n.d.), and via modeling (Anderson et al. 2024). Temperature dependent resource storage may arise when resource uptake and resource assimilation occur at rates which depend differently on temperature, for example, if resources are taken up more rapidly than they are assimilated while within a certain range of cool temperatures (Anderson et al. 2024). Then, when the cells are exposed to warm conditions, they can use these stored resources to divide more rapidly than cells acclimated to warm conditions (Layden et al. 2022). When these resources run out, cells proceed slowly with acclimation to the new temperature, as cells suddenly exposed to cool conditions do, perhaps by generating heat shock proteins, as observed in algae (Layden et al. 2022; Magni et al. 2018). For the resource storage hypothesis to be applicable to protists, cells may experience a change to the number or size of cellular food storage in vacuoles given changes in temperature, storing more consumed food in vacuoles in cool conditions than warm conditions. However, one recent study demonstrated that a marine ciliated protist in warming conditions does not exhibit altered stoichiometry, suggesting that cooler temperatures may not always lead to nutrient enrichment in our system (Calbet and Saiz 2022).

A simpler explanation for the observed detrimental effect of acclimation to warm conditions may be cumulative thermal stress. The thermal optimum of *Colpidium striatum* is approximately 20°C, though it exhibits positive intrinsic growth rates up to 28°C, meaning that our experimental temperature of 26°C may have been stressful. As observed in microbes, plants, and other organisms (Bigelow 1921; Neuner and Buchner 2023), heat can become increasingly stressful over time due to protein denaturation, possibly causing slower growth the longer a population is subjected to these conditions. Heat stress may have caused the populations which were acclimated to 26°C to grow more slowly than populations acclimated to 15°C in both experimental temperatures. Additionally, cumulative heat stress may explain the decline in growth rate over increasingly prolonged acclimation to 26°C in the short‐term exposure experiments and perhaps the increased frequency of extinction among acclimated populations at 26°C in the long‐term exposure experiments. This hypothesis could be tested by extending the number of acclimation days used as treatments in the short‐term exposure experiment to ten or more, clarifying the trajectory of acclimation after 5 days.

### 4.3 Limitations and Considerations

It is unlikely that sexual reproduction and adaptation meaningfully influenced our results. While ciliates mainly reproduce asexually, there is a very slight possibility that *Colpidium* cells engaged in conjugation, trading genetic material, since the stock cultures may have contained multiple mating types. In theory, conjugation could enable adaptation (Plebani 2015), which could be confounding our results. However, conjugation was never observed while counting cells and is extremely uncommon under our culture conditions. Moreover, our estimations of acclimation effects are based on data from only a few days, which is likely too short a time frame for significant adaptation to have taken place. Similar studies which adapted protists to various temperatures, even very rapidly, utilized many more generations than our longest experiments, and especially more generations than the first few days of experiments used here to estimate intrinsic growth rates (Plebani 2015; Weber de Melo et al. 2020). It is unlikely that our experimental conditions provided strong enough selection and large enough standing genetic variation to enable extremely rapid evolution over the only four to ten generations in the first week of experiments. Additionally, stock populations used for the final experiments that we conducted exhibited acclimation similar to the first experiments conducted.

While we initially considered 7 days of acclimation sufficient for “full acclimation,” the results of the short‐term exposure experiment revealed that *Colpidium*'s process of acclimation to warm conditions could possibly take more than 5 days. Therefore, populations at the beginning of experiments could have been at various intermediate stages of acclimation in the different experimental blocks. However, of the two experiments, only the first experimental block of the long‐term exposure experiment began with *Colpidium* acclimated for 7 days. All other experimental blocks began with more than 10 days of acclimation.

All microcosms began with the same density of bacterial prey, but it is possible that bacterial growth rates and abundance meaningfully varied with temperature, thereby affecting ciliate growth rates. The strains used were cultured from stock populations raised at room temperature, which could have had their own complex responses of growth rate to thermal variation. If the bacterial growth rate were low enough under some conditions to limit protist growth rates, this could have contributed to the effects of acclimation that we observed. Without direct measurement of bacterial abundances under various thermal regimes, we cannot rule out prey limitation as a contributor to our findings, and this possibility could be pursued in future experiments.

Our results and inferences are based on experiments conducted in only two temperatures near the extremes of *Colpidium*'s thermal tolerance range, which may have influenced some results. In future, protist microcosms could be constructed with populations acclimated to and grown in at least one intermediate temperature, for example, 20°C, which would provide information regarding a broader set of possible hypotheses (Huey et al. 1999). Luhring and Delong (2017) and Fey et al. (2021) pursued this approach with 48‐h and at least 2‐week acclimation periods, respectively, for each acclimation treatment. If populations were acclimated to a range of temperatures for varying amounts of time, thermal performance curves (TPCs) could be developed for each day of acclimation to each temperature. In this way, one could construct TPCs for each possible recent thermal history, allowing for detailed modeling of population dynamics in variable thermal environments. Or, with less experimental effort, the approach recently developed by Payne et al. (2025) could be applied, a method based on the temperature dependence of metabolic rate. Their method could predict effects of acclimation on heat tolerance given any prior acclimation temperature from the heat tolerance of ciliates with one acclimation temperature by using a conceptual “metabolic currency” which accumulates over time during heating.

## 5 Conclusions

The intrinsic growth rates of ciliated protists depend on both acclimation and the direction of thermal change. Acclimation to cool conditions increases growth rates in warm conditions relative to populations acclimated to warm conditions. Acclimation to cool conditions appears to take approximately 3–5 days while the duration of acclimation to warm conditions remains unclear. Demographic rates are sensitive to acclimation, and the dynamics of these effects depend on both prior and present temperature, with complex effects on the dynamics of populations and likely higher levels of organization in thermally variable environments.

## Author Contributions

**Julia Bebout:** conceptualization (equal), data curation (lead), formal analysis (lead), investigation (lead), methodology (equal), software (lead), writing – original draft (lead), writing – review and editing (equal). **Jeremy W. Fox:** conceptualization (equal), funding acquisition (lead), methodology (equal), writing – review and editing (equal).

## Funding

This work was supported by a Natural Sciences and Engineering Research Council of Canada Discovery Grant to J.W.F.

## Conflicts of Interest

The authors declare no conflicts of interest.

## Data Availability

Data and code are available on Dryad at the following link: https://doi.org/10.5061/dryad.3ffbg79tf.
